# Effects of thriving at work on employees’ family role performance: A moderated mediation model

**DOI:** 10.3389/fpsyg.2023.1079201

**Published:** 2023-03-07

**Authors:** Baoyan Yang, Shaoqing Su, Zhaobiao Zong, Qiaoqiao Du, Junyi Wang

**Affiliations:** ^1^School of Psychology, Northwest Normal University, Lanzhou, Gansu, China; ^2^School of Psychology and Cognitive Science, East China Normal University, Shanghai, China

**Keywords:** thriving at work, family role performance, work–family enrichment, family-supportive supervisor behavior, work–home resource model

## Abstract

**Objective:**

Existing research has demonstrated that thriving at work has a positive effect on work performance, but little is known about how thriving at work affects family role performance. Based on the work–home resource model, this study examines the impact mechanism of thriving at work on family role performance.

**Methods:**

This paper uses an experience sampling method to conduct a 5-day daily study of 151 married employees in Northwest China, and the data were analyzed using a multilevel linear model.

**Results:**

We find that thriving at work positively affects family role performance partly through the mediating effect of work–family enrichment at the individual level. Moreover, family-supportive supervisor behavior moderates the relationship between thriving at work and work–family enrichment. Through work–family enrichment, family-supportive supervisor behavior also moderates the indirect relationship between thriving at work and family role performance. Specifically, the higher the level of family-supportive supervisor behavior, the stronger the indirect effect of thriving at work on family role performance through work–family enrichment.

**Conclusion:**

Previous research has focused more on the effects of thriving at work within the work domain, suggesting that thriving at work can have a positive impact on work outcomes. However, only a few studies have examined the positive relationship between thriving at work and family role performance from the perspective of employees’ positive psychological resources. This paper explores the positive effects of thriving at work on family role performance based on a resource flow perspective and identifies its potential boundary conditions. This study enriches the theoretical research on the relationship between thriving at work and family role performance. Additionally, it provides a new foothold and research perspective on improving work–family enrichment.

## Introduction

Work and family are two crucial parts of life. People work hard to achieve career development and improve their family’s quality of life. However, in most cases, it is difficult for people to balance work and family. Therefore, achieving career development and family happiness simultaneously is not only a problem of individual concern but also an important issue that academia and managers have been concerned about for a long time (Jachimowicz et al., 2021; Lin et al., 2021). In the Chinese TV series *The Perfect Mate*, the heroine gave up the job she loves to take care of her family, then realized that being a housewife is not for her and goes back to her career, where her experiences at work make her more aware of how to run her family life, and finally lives the life she wants. In accordance with this TV series, existing research also confirms that the level of engagement demonstrated at work has an impact on work–family balance and family satisfaction through positive work events that individuals share with their spouses at home (Ilies et al., 2017). Therefore, it is possible to achieve both work and family life balance, but the key is to find mechanisms through which these two aspects can be mutually reinforced.

**Figure 1:**
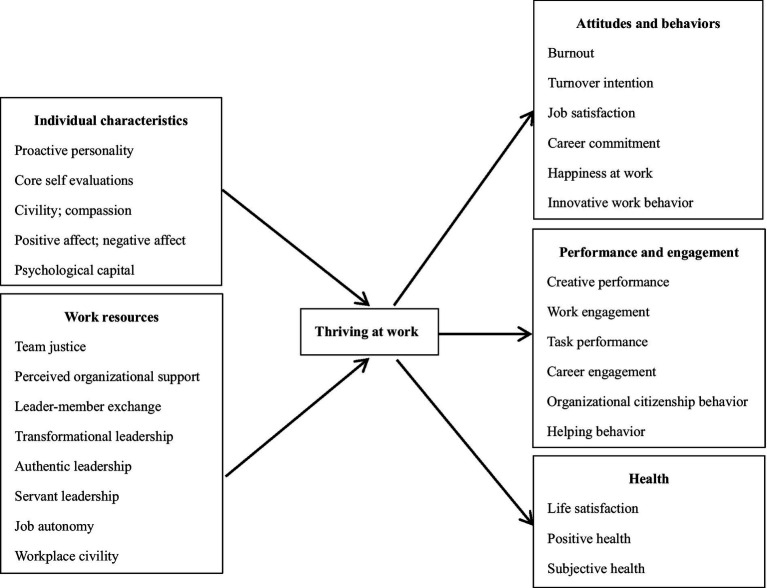
Antecedents and consequences of thriving at work.

Thriving at work refers to the positive psychological state of learning and the vitality that accompanies an individual’s work process (Spreitzer et al., 2005; Ren et al., 2022). Previous studies have focused more on the effects of thriving at work in the workplace. It is believed that thriving at work can effectively alleviate negative attitudes and behaviors that occur at work (Abid et al., 2016, 2018; Hildenbrand et al., 2018; Chang and Busser, 2020; Jiang et al., 2020, 2021; Qaiser et al., 2020; Abid and Contreras, 2022), enhance work-related performance and engagement (Abid et al., 2018; Frazier and Tupper, 2018; Marchiondo et al., 2018; Christensen-Salem et al., 2021; Jiang et al., 2021), and promote employee mental health levels (Walumbwa et al., 2018; Kleine et al., 2019; Zhai et al., 2020; Rehmat et al., 2021). In addition, some scholars have suggested that personal characteristics (Porath et al., 2012; Jiang, 2017; Walumbwa et al., 2018; Kleine et al., 2019; Chang et al., 2020; Elahi et al., 2020; Abid and Contreras, 2022) and job-related resources within the work domain (Mortier et al., 2015; Abid et al., 2016; Niessen et al., 2017; Walumbwa et al., 2018; Bensemmane et al., 2019; Zhang et al., 2019; Jiang et al., 2020) are predictive of thriving at work (see Figure 1 for details on the antecedent and outcome variables of thriving at work).

In recent years, some scholars have started to study the positive relationship between family life and thriving at work, but such studies are limited to the impact of family life and family-related policies on thriving at work, and fewer scholars have focused on the positive effect of thriving at work on family life (Carmeli and Russo, 2016). In today’s society, people pay more attention to the quality of life, family needs, and the harmonious development of work and family (Tariq and Ding, 2018). Therefore, it is necessary to discuss and test the possible mechanisms through which the positive state of work (e.g., thriving at work) contributes to the quality of life of employees’ families (Amstad et al., 2011; Jia et al., 2014).

Work–family enrichment is the extent to which an individual’s experience in a work role contributes to improving the quality of their family’s life (Greenhaus and Powell, 2006). Resources representing one domain help develop personal resources that drive increased outcomes in another domain (Ten Brummelhuis and Bakker, 2012). The work–home resources model uses personal resources as a link between resources in one domain and outcomes in another domain, systematically explaining the causal logic behind work–home enrichment that are most likely to occur and the developmental processes (Ten Brummelhuis and Bakker, 2012; Ten Brummelhuis and Greenhaus, 2018). For example, work–family enrichment occurs when resources from the work domain increase personal resources and are used to improve outcomes in the family domain.

Enrichment is formed when the resources that individuals accumulate in their thriving at work allow them to perform better in their family life. The formation of resources is a key driver of the enrichment process as they are an asset that individuals need to draw upon when faced with problems (Wayne et al., 2007). Specifically, family role performance refers to the results of fulfilling role-based obligations and expectations when individuals participate in family activities (Chen et al., 2014; Derks et al., 2016). If thriving at work brings many positive resources to an individual, then these resources can be applied to the family to improve the quality of family life; thus, work–family enrichment should be one of the necessary conditions and critical links in this positive effect. Therefore, this study explores this issue and verifies the possible mediating role of work–family enrichment in the relationship between thriving at work and family role performance using a work–home resource model.

In addition, the ability of employees to thrive at work is often tied to their supervisor’s interpretation and implementation of the organization’s family support culture. A supervisor who cares about the needs of employees’ families can create a family-supportive organizational environment for employees to feel supported by the organization, which can contribute to the acquisition and development of individual resources (Ten Brummelhuis and Bakker, 2012). A growing number of studies have also demonstrated that family-supportive supervisor behavior is one of the key situational factors that affect the positive spillover between work and family (Russo et al., 2018; Straub et al., 2019). Family-supportive supervisor behavior refers to family support behaviors that leaders demonstrate to employees to assist them to assume family role responsibilities and obligations, which are aimed at helping employees better fulfill their work and family responsibilities and improving the relationship between work and family (Hammer et al., 2009; Booth-LeDoux et al., 2020). Studies have revealed that when the behavior of a family-support supervisor is high, employees tend to be more energetic, better able to balance work and family, and have higher family happiness (Matthews et al., 2014; Zhang and Tu, 2018). Thus, family-supportive supervisor behavior plays an important role in how thriving at work influences family role performance through work–family enrichment. Based on the work–home resource model, we propose that family-supportive supervisor behavior moderates the indirect relationship between thriving at work and family role performance through work–family enrichment.

In summary, based on the work–home resource model, this research explores the mechanism of work–family enrichment and family-supportive supervisor behavior in the process of thriving at work, which affects family role performance. Recent studies have recognized that, as an emotion-cognitive state, individuals’ thriving at work is volatile. Thus, employees’ thriving at work may vary during the working day because of various internal and external factors (Niessen et al., 2012; Kleine et al., 2019). Therefore, this study intends to use an experience sampling method to explore how changes in employees’ daily thriving at work affect their daily family role performance.

## Theory and hypotheses

### Thriving at work and family role performance

Thriving at work is a positive psychological state, so when employees invest more energy and learn new skills at work, they tend to feel a higher sense of achievement, which is conducive to self-efficacy and happiness in life. They then bring this positive emotional experience to the family, which increases sharing and communication with their partners and helps them to better perform their family functions (Ilies et al., 2017; Straub et al., 2019). Moreover, after a busy day at work, employees may feel that they have put in a lot of effort and performed to the best of their ability and are motivated to go home and focus more on family life and handling family matters (Kahn, 1990). Therefore, we infer that thriving at work is positively related to employees’ family role performance.

The work–home resource model provides a theoretical basis for understanding the positive effects of thriving at work on employee family role performance. The model is a theory that systematically explains the positive interaction between work and family by describing the processes and conditions under which individual resources link resources in one domain to outcomes in the other. According to the work–home resource model (Ten Brummelhuis and Bakker, 2012), the effects of each domain will spill over from one to the other, despite the temporary separation between work and family (Wood et al., 2020). Therefore, when employees are highly thriving at work, the experience and resources in their work can significantly help them better solve related problems in the family and improve their efficiency and performance (Ilies et al., 2017). In addition, studies have found that individuals who highly thrive at work tend to have higher emotional management ability at work. Through effective emotional management, individuals can avoid negative emotions and events, reduce the depletion of psychological resources, and increase their ability and motivation to fulfill family obligations and play family roles (Greenhaus and Powell, 2006; Zheng and Powell, 2012). Thus, we propose the following hypothesis:

*Hypothesis 1*: Thriving at work has a significant positive effect on family role performance.

### The mediating role of work–family enrichment

The mechanisms through which thriving at work affects employees’ outcomes in the family domain are explored through the lens of work–family enrichment. Work–family enrichment is when resources in one domain “contribute to the development of personal resources that drive increased outcomes in another domain” (Ten Brummelhuis and Bakker, 2012). Through an instrumental approach, research has found that thriving at work can affect employees’ work–family enrichment. Employees who highly thrive at work are motivated by internal factors, are enthusiastic about work, and have a stronger ability to work and learn, which are conducive to acquiring more skills and gaining more opportunities to accumulate knowledge. This helps employees to perform better at home and achieve work–family enrichment (Bakker et al., 2012; Ilies et al., 2017). In addition, thriving at work enhances work–family enrichment in emotional ways. As mentioned earlier, thriving contains an element of vitality, which is a positive psychological experience. Research has demonstrated that thriving at work is associated with work resources that reduce work stress and stimulate positive emotions (Xanthopoulou et al., 2007). Employees who highly thrive at work tend to have high positive emotions at work, and such emotional feelings (also a type of resource) spill over to the field of family life, which enables employees to have positive emotions when playing family roles, thereby enhancing work–family enrichment (Ilies et al., 2017). Therefore, we propose the following hypothesis:

*Hypothesis 2*: Thriving at work has a significant positive effect on work–family enrichment.

Work–family enrichment leads to the mutual transfer of resources in different fields and helps individuals acquire more resources. When individuals have more disposable resources, they can deal with the pressure of family life and achieve family role performance (Russo et al., 2018; Wu et al., 2021). Studies have found that work–family enrichment, which is a positive result of resources accumulated by employees in the process of work and brought into family life, can generate more resources in the family, and the motivation level stimulated by resource accumulation makes individuals perform better in the family (Lazarova et al., 2010). Other studies have confirmed that work–family enrichment positively promotes individuals’ physical and mental health, and good physical and psychological resources increase the possibility of role involvement, thus positively affecting family role performance (Williams et al., 2006; Rich et al., 2010). The study of Ma et al. (2014) also found that when employees have a high work–family enrichment, they often have a high level of family involvement, which further promotes family role performance. Thus, we propose the following hypothesis:

*Hypothesis 3*: Work–family enrichment has a significant positive effect on family role performance.

Combining *H2* and *H3*, we propose the following:

*Hypothesis 4*: Work–family enrichment plays a mediating role in the relationship between thriving at work and family role performance.

### The moderating effect of family-supportive supervisor behavior

The work–home resource model assumes that the interaction between multiple resources (e.g., psychological and situational) can have a synergistic effect (Greenhaus et al., 2012). According to this model, contextual resources (e.g., pro-family policies and family-supportive supervisor behavior) play a moderating role in the relationship between individual psychological resources stimulated by the work domain and the work–family facilitation relationship. Hobfoll et al. (2018) suggested that by cultivating valuable work resources, managers can create a good cycle of resources for their employees. Family-supportive supervisor behavior is supportive behavior exhibited by leaders toward employees’ family life, constructing a person-centered, pro-family style of leadership to influence employees’ attitudes and behaviors, which help them to better fulfill their work and family responsibilities and improve the relationship between work and family. Specifically, with high family-supportive supervisor behavior, individuals receive more resource support from their organization and accumulate a wealth of personal resources. At this point, they are more likely to focus on resource acquisition and pursue access to resources (Halbesleben et al., 2014). Therefore, when employees receive a high level of encouragement and support from their leaders, they will expand their work resources and perceive more energy at work, which would effectively contribute to their work–family enrichment and enhance their problem-solving skills and fulfillment of family roles in the family domain. Research has also found that when leaders provide employees with instrumental support, employees usually concentrate on their work and make full use of resources to achieve the sharing and mutual promotion of resources in the field of work and family (Matthews et al., 2014; Tang et al., 2017).

However, with low family-supportive supervisor behavior, an individual faces a greater threat to resources and workload exerts progressively more pressure or hindrance on the individual, which may inhibit the individual’s current psychological resources (thriving at work), and it prevents the positive role it plays from being fully realized and utilized (Hobfoll, 2011). Therefore, when experiencing low levels of family-supportive supervisor behavior, employees are prevented from further depleting their own resources for thriving at work by experiencing a lack of total contextual resources, thereby reducing the strength of the positive relationship between thriving at work and work–family enrichment. For example, studies have revealed that employees with low levels of family-supportive supervisor behavior are more likely to have a strong sense of time encroachment and habitually define themselves as “outsiders,” which impedes the flexible transition between work and family roles (Wang et al., 2018). Thus, we propose the following hypothesis:

*Hypothesis 5*: Family-supportive supervisor behavior plays a moderating role in the relationship between thriving at work and work–family enrichment. The higher the family-supportive supervisor behavior, the stronger the positive effect of thriving at work on work–family enrichment.

The above discussion reveals that family-supportive supervisor behavior moderates the relationship between thriving at work and work–family enrichment and influences the indirect effect of thriving at work on family role performance through work–family enrichment. Specifically, work–family enrichment mediates the effect of thriving at work on family role performance, but the level of family-supportive supervisor behavior influences the strength of the mediating effect. Based on the above reasoning, we propose the following hypothesis:

*Hypothesis 6*: Family-supportive supervisor behavior moderates the indirect effect of thriving at work on family role performance through work–family enrichment. The higher the family-supportive supervisor behavior, the stronger the indirect effect of thriving at work on family role performance through work–family enrichment.

**Figure 2:**
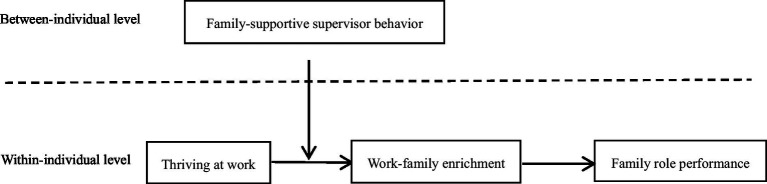
The proposed theoretical model.

The theoretical model of this study is summarized in Figure 2.

## Methods

This research conducts a questionnaire survey on married employees of four enterprises and public institutions in northwest China. The enterprises are in the following industries: medicine and health, education and training, as well as science and technology about agriculture and forestry. To deeply explore the changes and feelings of employees in a natural environment, increase the inference of the causality of the variables, and reduce common method biases, this study employs the experience sampling method to collect data. With the support and cooperation of senior managers and human resources departments, the researchers invited 478 front-line employees from four companies to participate in the survey, among which 200 employees volunteered to participate in the survey. The theoretical model of this study has variables at both within- and between-individual levels. Because many respondents do not answer online questionnaires seriously, such as by filling the questionnaire several times or not answering some questions, a paper questionnaire is adopted in this study.

The research team placed the questionnaires in small, sealable, and anonymous disposable envelopes, each with double-sided tape that was pre-laminated. Before the survey, the supervisors of each company emphasized the importance of the research in helping the surveyed employees to thrive at work, explained the research process in detail, expressed their support for the project, and encouraged everyone to cooperate. The supervisor then handed out one-time questionnaire envelopes to the subjects and left the room afterward. The research team stayed in the meeting room and collected the questionnaires after completion. Thus, data on between-individual level variables (demographic variables, intrinsic motivation, and family-supportive supervisor behavior) were collected.

The data about the following within-individual level variables were collected over five consecutive working days: thriving at work, work–family enrichment, family role performance, and job demands. The questionnaires were distributed during working hours and at night. (1) Distribution during working hours: the supervisor distributes the sealable questionnaire about thriving at work and job demands to the respondents to fill at regular intervals (4:30 PM on weekdays) and leaves the room afterward. To ensure the timeliness of the survey, the responses were collected on the spot by the research team. (2) Distribution in the evening: The questionnaires about family role performance and work–family enrichment were packed into file bags and distributed to the respondents before leaving work, and the respondents were instructed to answer them carefully from 20:30 to 21:00. The research team reminded the subjects who volunteered to participate in the WeChat group to fill it out in time. The next morning, the subjects returned the sealed envelopes to the research team. In addition, the one-time and daily questionnaires were paired using a coded format (i.e., the one-time questionnaires were numbered and subjects were asked to remember their number, and on five consecutive days, the subjects were asked to fill in their questionnaire number). The data collection process was completely anonymous and voluntary. The researcher gave a small gift to respondents who completed the questionnaire survey during the five working days.

In the first stage, 200 questionnaires were distributed; 191 were retrieved, and 178 were valid. In the daily survey phase, 890 questionnaires were collected from 178 married employees, with 755 valid questionnaires—an effective rate of 85.1%. According to the descriptive statistics, 31 respondents work in science and technology about agriculture and forestry 1 (20.5%) industry, 41 in medicine and health (27.2%), 43 in education and training (28.5%), and 36 in science and technology about agriculture and forestry 2 (23.8%). In terms of gender, 74 are males (49.0%), and 77 are females (51.0%). In terms of age, 86 (57.0%) are under 35 years, 42 (27.8%) are 36–45 years, and 23 (15.2%) are more than 45 years. Regarding education, 42 (27.8%) have a junior college degree or below, and 109 (72.2%) have a bachelor’s degree or above. Regarding working experience, 40 (26.5%) have worked for 5 years or less; 48 (31.8%) have worked for 5–10 years, and 63 (41.7%) have worked for 10 years or more.

### Variables

This study measures job demands on a five-point Likert scale, with 1 representing “very small” and 5 representing “very large.” Other variables are measured on a seven-point Likert scale, with 1 representing “strongly disagree” and 7 representing “strongly agree.” To ensure that the meaning of the translated Chinese questionnaire is consistent or similar to that of the original English questionnaire, members of the research group and the English language professionals were engaged to translate the questionnaire. Due to the particularity of the experience sampling method, we modify the original scale used in the five consecutive days of work by adding the time description qualifier of “today.”

Thriving at work. The scale of thriving at work has two dimensions—vitality and learning. Studies have been conducted to evaluate the vitality and learning dimensions in an integrated manner and data were obtained through subjective evaluations, and this paper also draws on previous practices to measure thriving at work (Riaz et al., 2018; Feeney and Fitzgerald, 2019; Li et al., 2020). The vitality dimension adopts the scale developed by Atwater and Carmeli (2009). It contains eight items, such as “Today, I feel active and energetic at work.” The learning dimension adopts the scale developed by Carmeli and Spreitzer (2011), containing three items, such as “Today, to what extent do the things you learn at work help you in your life?” As the current mainstream measurement instrument, the reliability of the scale has been validated in many research measurements with samples involving members of different types of organizations, which shows that the scale has a relatively wide applicability (Niessen et al., 2012; Kleine et al., 2019; Lin et al., 2020). In the present study, Cronbach’s alpha of the vitality dimension is 0.86, and that of each of the 5 days ranges from 0.84 to 0.87. Cronbach’s alpha of the learning dimension is 0.74, and that of each of the 5 days ranges from 0.70 to 0.80. Total scale Cronbach’s alpha is 0.89, and that of each of the 5 days ranges from 0.88 to 0.91.

Family role performance. We use the scale developed by Chen et al. (2014) to measure family performance. It contains eight items, including “I will maintain things around the home after work today.” The reliability of the scale has been validated in previous studies and has wide applicability (Las Heras et al., 2017; Kelly et al., 2020). Its Cronbach’s alpha is 0.92, and that of each of the 5 days ranges from 0.89 to 0.93.

Work–family enrichment. We use the scale developed by Wayne et al. (2004) to measure work–family enrichment. It contains four items, including “The things I do at work help me deal with personal and practical issues at home.” After the development of this scale, the reliability and validity of the scale were tested using empirical data and analysis, and the results were more than satisfactory for further research and analysis of the work and family–related topics (Li et al., 2022). Its Cronbach’s alpha is 0.80, and that of each of the 5 days ranges from 0.78 to 0.85.

Family-supportive supervisor behavior. We use the scale developed by Hammer et al. (2009) to measure family-supportive supervisor behavior. It contains four items. Hammer et al. (2009) argued that family-supportive supervisor behavior is mainly reflected by a leader’s supportive behavior for employees’ family life and other aspects. The strength of its effectiveness mainly comes from employees’ perception, so employees should fill out the questionnaires. The items include “My supervisor and I can talk effectively to solve conflicts between work and nonwork issues.” After the development of this scale, the reliability and validity of the scale were tested using empirical data and analysis, and the results were more satisfactory for further research and analysis (Erdogan et al., 2022; Yu et al., 2022). Its Cronbach’s alpha is 0.79.

Control Variables. This study controls for demographic variables that can affect the results, such as sex, age, education level, and years of service. Moreover, previous studies have found that job demands can affect work–family relationships (Bakker et al., 2008), and intrinsic motivation can affect thriving at work (Menges et al., 2017). Therefore, these within-individual variables are also controlled in this study. The scale developed by Karasek (1979) is used to measure job demands; the items include “Requires working fast.” Its Cronbach’s alpha is 0.87, and that of each of the 5 days ranges from 0.85 to 0.89. Intrinsic motivation is measured by the scale developed by Ryan and Connell (1989); the items include “Because I enjoy the work itself.” Cronbach’s alpha of intrinsic motivation is 0.86.

### Data analysis

Maas and Hox (2005) suggested that a sample size of more than 50 people, collected for five consecutive days, is a relatively accurate data estimate. The final valid data for this study were data on 151 people, collected for five consecutive days, which meet the minimum sample size requirement. This suggests that the data are suitable for multilevel analysis. In terms of specific analysis methods, correlation and reliability analyses were carried out using SPSS. In addition, the data had a relatively obvious nested structure, i.e., the measurement level was nested within the individual level, so Mplus 8.4 was used to conduct multilevel validation factor analysis and multilevel modeling to test the research hypotheses. First, following the suggestions of Hofmann and Gavin (1998) and Enders and Tofighi (2007), all within-individual level variables are centralized with group-mean centering to effectively exclude the influence of between-individual variable differences. Therefore, the results of the data analysis fully reflect the relationship between within-individual differences. Second, all between-individual level variables are centralized with the grand-mean centering. Moreover, the Parametric Bootstrap program (20,000 Monte Carlo replicates) recommended by Preacher et al. (2010) is used to test the intermediate effect by estimating the bias correction confidence interval at 95%. The moderating effect is analyzed using the random slope method, and the moderating effect is tested using the Monte Carlo Simulation (MCS) method.

## Results

### Descriptive statistical analysis

This study examines the percentage of intra-individual variance and the coefficient of variation between groups (ICC1) for intra-individual level variables separately. The results reveal that all these variables have sufficient intra-individual variance percentages (see Table 1). Therefore, thriving at work [ICC(1) = 0.37, *F*(150, 604) = 3.99, *p* < 0.001], work–family enrichment [ICC(1) = 0.40, *F*(150, 604) = 4.38, *p* < 0.001], and family role performance [ICC(1) = 0.47, *F*(150, 604) = 5.547, *p* < 0.001] are suitable for cross-layer analysis. The mean, standard deviation, and correlation coefficient of the variables are presented in Table 2. Among the within-individual variables, thriving at work and work–family enrichment (*r* = 0.27, *p* < 0.001) have a significantly positive correlation with family role performance (*r* = 0.33, *p* < 0.001). Work–family enrichment and family role performance (*r* = 0.40, *p* < 0.001) have a significantly positive correlation. This lays a preliminary foundation for testing the hypotheses. Moreover, job demand is significantly negatively correlated with thriving at work (*r* = −0.07, *p* = 0.049) and family role performance (*r* = −0.10, *p* = 0.009). Thriving at work is positively correlated with family-supportive supervisor behavior (*r* = 0.24, *p* = 0.003) and intrinsic motivation (*r* = 0.18, *p* = 0.027). Family-supportive supervisor behavior is positively correlated with work–family enrichment (*r* = 0.31, *p* < 0.001) and intrinsic motivation (*r* = 0.17, *p* = 0.038).

**Table 1:** Percentage of variance of within-individual variables.

Variable	Within-individual variance (*e^2^*)	Between-individual variance (*r^2^*)	Percentage of within-individual variance (*%*)
Thriving at work	0.48	0.28	63.16%
Work–family enrichment	0.87	0.58	60.00%
Family role performance	0.89	0.80	52.66%

Percentage of within-individual variance = within-individual/(within-individual + between-individual variance).

**Table 2:** Means, standard deviations, and correlations of the study variables.

Between-individual level variables	M	SD _within_	SD _between_	1	2	3	4	5	6	7	8	9	10
1. Sex	0.51	—	0.50	1									
2. Age	1.58	—	0.74	−0.12	1								
3. Educational status	0.72	—	0.45	0.07	−0.09	1							
4. Work experience	2.15	—	0.81	−0.24**	0.58**	0.06	1						
5. Intrinsic motivation	4.47	—	1.27	0.01	0.05	0.05	0.03	1					
6. Family-supportive supervisor behavior	4.61	—	1.16	−0.09	−0.04	−0.14	−0.03	0.17*	1				
*Within-individual level variables*													
7. Thriving at work	4.82	0.87	0.62	−0.16*	−0.10	0.01	0.08	0.18*	0.24**	1	0.27***	0.33***	−0.07*
8. Work–family enrichment	4.61	1.20	0.87	−0.03	−0.08	0.06	0.02	0.16	0.31**	0.37**	1	0.40***	0.02
9. Family role performance	4.96	1.30	0.99	−0.13	−0.06	0.12	0.03	0.04	0.01	0.34**	0.37**	1	−0.10**
10. Job demands	4.08	0.93	0.77	−0.14	0.25**	0.03	0.06	0.00	0.12	−0.12	−0.03	−0.25**	1

(1) Between-individual level variables = 151; Within-individual level variables = 755. (2) The result below the between-individual level correlation coefficient is the correlation coefficient calculated by aggregating within-individual level variables to between-individual level variables. (3) Male = 0; Female = 1; under the age of 35 = 1; under the age of 36 to 45 = 2; above the age of 45 = 3; junior college and below = 0; bachelor degree or above = 1; 5 years or less working experience = 1; 5 to 10 years of working experience = 2; more than 10 years of working experience = 3. (4) *p < 0.05, **p < 0.01, ***p < 0.001.

### Confirmatory factor analysis

We use Mplus 8.0 to conduct a multi-level confirmatory factor analysis (MCFA) of our focal variables to verify discriminant validity. The results reveal that the four-factor model fits best (*χ^2^/df* = 1.95, RMSEA = 0.04, SRMR_within_ = 0.0, SRMR_between_ = 0.01, CFI = 0.97, TLI = 0.97), and its fitting coefficients are better than those of other models (see Table 3), indicating that there is good discriminative validity among the variables.

**Table 3:** CFA.

Model	χ^2^	df	*χ*^2^/df	Δ*χ*^2^/(Δdf)	CFI	TLI	RMSEA	SRMR within	SRMR between
Four-factor model: A; B; C; D	447.24	229	1.95	–	0.97	0.97	0.04	0.03	0.01
Three-factor model: A+ B; C; D	1304.20	231	5.65	856.96(2)	0.86	0.84	0.08	0.09	0.01
Two-factor model: A + B + C; D	3381.93	232	14.58	2934.69(3)	0.59	0.54	0.13	0.14	0.01
One-factor model +CMV: A; B; C; D; CMV	364.45	206	1.77	–	0.98	0.97	0.03	0.03	0.01

(1) Within-individual level variables = 755; (2) A = thriving at work, B = work–family enrichment, C = family role performance, D = family-supportive supervisor behavior; (3) “+” indicates the combination of two factors into one factor; (4) CMV represents common method bias; (5) all Δ*χ*^2^ in *p* < 0.001significant.

Podsakoff et al. (2003) also suggested that the Harman single factor and controlling for an unmeasured single latent methods factor should be used to evaluate common method biases. The results reveal that in the unrotated factor analysis, the variance explanation rate of the first common factor is 27.57%, which is lower than 40%. Common method factors (*χ*^2^/*df* = 1.77, RMSEA = 0.03, SRMR_within_ = 0.03, SRMR_between_ = 0.01, CFI = 0.98, TLI = 0.97) are added to compare the fitting of the four-factor model (see Table 3), and the results reveal that the fitting indices—CFI, TLI, RMSEA, SRMR_within_, and SRMR_between_—do not significantly improve. Therefore, common method biases have little impact in this study.

### Test of hypotheses

Table 4 reveals that the zero model of work–family enrichment and family role performance is a random effect one-way ANOVA without any variables; Models 1 and 5, on the basis of the zero model, add within-individual control variables and between-individual control variables; Models 2 and 6, on the basis of Models 1 and 5, add the within-individual level variable thriving at work; Model 3, on the basis of Model 2 by adding the between-individual level moderating variable family-supportive supervisor behavior; Model 4 by adding an interaction term between thriving at work and family-supportive supervisor behavior to Model 3; and Model 7 by adding the within-individual level variable work–family enrichment to Model 6.

**Table 4:** Results of the multi-level analysis.

Variables		Work–family enrichment					Family role performance			
		Zero model	M1	M2	M3	M4	Zero model	M5	M6	M7
	Intercept	4.61***	3.87***	3.87***	3.94***	3.92***	4.96***	4.29***	4.28***	4.42***
Within-individual control variables	Job demands		0.13*	0.13*	0.12*	0.09		0.22***	0.23***	0.19***
Between-individual control variables	Science and technology about agriculture and forestry 1		−0.54*	−0.54*	−0.53	−0.49		−1.20***	−1.21***	−1.16***
	Medicine and health		−0.34	−0.34	−0.33	−0.30		−0.99***	−1.00***	−0.96***
	Education training		−0.28	−0.28	−0.24	−0.24		−0.07	−0.07	−0.07
	Sex		−0.02	−0.02	0.02	0.01		−0.30*	−0.30*	−0.31*
	Education background		0.02	0.02	0.11	0.12		0.22	0.21	0.22
	Age		−0.20	−0.20	−0.16	−0.16		0.04	0.04	0.04
	Working years		0.11	0.11	0.10	0.10		−0.03	−0.03	−0.04
	Intrinsic motivation		0.12*	0.12*	0.09	0.09		0.07	0.07	0.07
Within-individual level	Thriving at work			0.23***	0.23***	0.20**			0.43***	0.34***
	Work–family enrichment									0.39***
Between-individual level	Family-supportive supervisor behavior				0.22***	0.22***				
Cross-layer interaction	Thriving at work*Family supportive supervisor behavior					0.27***				
Variance component	Within-individual variance (*e*)	0.87***	0.86***	0.84***	0.84***	0.61***	0.89***	0.86***	0.78***	0.65***
	Between-individual variance (*r_0_*)	0.58***	0.53***	0.53***	0.48***	0.52***	0.80***	0.62***	0.64***	0.66***
Model fit index	Logarithmic likelihood (LL)	−1128.61	−1120.89	−1112.30	−1105.93	−1058.44	−1154.56	−1130.99	−1098.84	−1044.69
	Freedom degree (*df*)	3	12	13	14	17	3	12	13	14
	Δ −2LL		15.43	17.18	12.75	94.97		47.14	64.30	108.31
	Δ *df*		9	1	1	3		9	1	1

(1) Between-individual level variables = 151; within-individual level variables = 755. (2) The coefficients in the table are non-standardized regression coefficients. (3) Other control variables are enterprises (agriculture and forestry about science and technology 2 is the reference group), sex, age, education training, and working years. (4) Robust maximum likelihood estimator (maximum likelihood). (5) When no control variables are included, the model still holds. (5) *p < 0.05, **p < 0.01, ***p < 0.001.

As presented in Table 4, in the family role performance model, the fit result of Model 5 significantly improves when control variables are added to the zero models (Δ − 2LL = 47.14, *p* < 0.001). The fit results of Model 6 significantly improve after adding thriving at work into the model (Δ − 2LL = 64.30, *p* < 0.001), and the regression coefficient between thriving at work and family role performance is 0.43 (*p* < 0.001), thus supporting *H1*.

As presented in Table 4, in the work–family enrichment model, the fit result of Model 1 does not significantly improve (Δ − 2LL = 15.43, *p* = 0.080) when the control variable is added to the zero models. The relevant result of Model 2 significantly improves (Δ − 2LL = 17.18, *p* < 0.001), and the regression coefficient between thriving at work and work–family enrichment is 0.23 (*p* < 0.001), supporting *H2*. The fit results of Model 3 significantly improve when family-supportive supervisor behavior is added to the model (Δ − 2LL =17.18, *p* < 0.001). The regression coefficient between family-supportive supervisor behavior and work–family enrichment is 0.22 (*p* < 0.001), and the regression coefficient between thriving at work and work–family enrichment is 0.23 (*p* < 0.001) but still significant.

Based on Model 6, the fit results of Model 7 significantly improve when work–family enrichment is added to the model (Δ − 2LL = 108.31, *p* < 0.001), and the regression coefficient between work–family enrichment and family role performance is 0.39 (*p* < 0.001), thus supporting *H3*. Moreover, because work–family enrichment partially explains family role performance, the regression coefficient between thriving at work and family role performance decreases to 0.34 (*p* < 0.001) but is still significant, suggesting that work–family enrichment may mediate the relationship between thriving at work and family role performance. To verify *H4*, we use the Monte Carlo method in the Parametric Bootstrap procedure recommended by Preacher et al. (2010) to test the mediation effect. The results of Table 5 indicate that the mediation effect of thriving at work on family role performance through work–family enrichment is 0.09, 95% CI [0.05, 0.13] excluding 0; the mediating effect accounts for 20.93% of the total effect. Thus, *H4* is supported.

**Table 5:** Mediating effect analysis.

Path	Effect size	95% CI
Total effect: Thriving at work→Family role performance	0.43	[0.32, 0.53]
Direct effect: Thriving at work→Family role performance	0.34	[0.25, 0.43]
Mediation effect: Thriving at work→Work–family enrichment→Family role performance	0.09	[0.05, 0.13]

**Figure 3:**
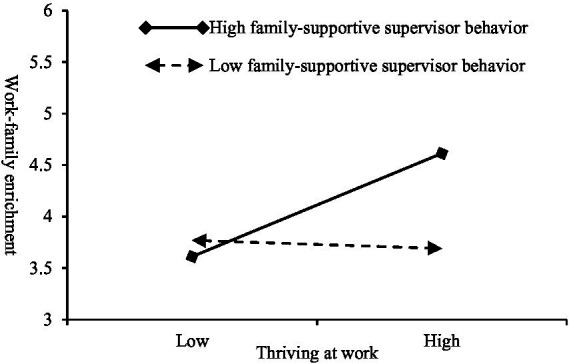
Moderating effect of family-supportive supervisor behavior on thriving at work and work–family enrichment.

Statistically, using the log-likelihood ratio test, we find that the chi-square statistic is significant (−2LL(1) = 79.84, *p* < 0.001). This implies that using random slope models provides a better fit than random intercept models. This study probes the cross-level moderating effect using a random slope analysis. Model 4 in Table 4 reveals that the interaction term between thriving at work and family-supportive supervisor behavior has a significant positive effect on work–family enrichment (*γ* = 0.27, *p* < 0.001). The results of the simple slope test (Figure 3) reveal that when the level of family-supportive supervisor behavior is high (1 SD above the mean), thriving at work has a significant positive effect on work–family enrichment (*γ* = 0.51, *p* < 0.001), and the prediction effect is relatively high. When the level of family-supportive supervisor behavior is low (1 SD below the mean), the effect of thriving at work on work–family enrichment is not significant (*γ* = −0.10, *p* = 0.353). Moreover, the difference between them is significant (*γ* = 0.61, *p* < 0.001), 95% CI [0.31, 0.92]. Thus, *H5* is supported.

To test the moderated mediation effect, we use the MCS method to analyze the mediating effect of work–family enrichment under different family-supportive supervisor behavior levels. As presented in Table 6, when the level of family-supportive supervisor behavior is high (1 SD above the mean), the indirect effect of thriving at work on family role performance through work–family enrichment is significant (indirect effect = 0.20, 95% CI [0.11, 0.29]). When the family-supportive supervisor behavior level is low (1 SD below the mean), the indirect effect of thriving at work on family role performance through work–family enrichment is not significant (indirect effect = −0.04, 95% CI [−0.13, 0.04]). Moreover, the difference between the two is significant (indirect effect = 0.24, 95% CI [0.12, 0.36]). Thus, *H6* is supported.

**Table 6:** Moderated mediation effects.

Path	Condition	Effect size	95% CI
Thriving at work→Work–family enrichment→Family role performance	High family-supportive supervisor behavior	0.20	[0.11, 0.29]
	Low family-supportive supervisor behavior	−0.04	[−0.13, 0.04]
	Difference	0.24	[0.12, 0.36]

## Discussion

This study found that thriving at work was a positive predictor of family role performance. The results of this study suggest that the two roles employees play in the work domain and family life are not always in contradictory opposition, and that the energy employees gain from participating in work activities helps to promote individual family role activities. While most previous studies have tended to emphasize the effects of thriving at work within the work domain (Porath et al., 2012; Hildenbrand et al., 2018; Chang and Busser, 2020; Christensen-Salem et al., 2021), we aim to explore how work affects employees’ family behaviors, which helps enrich the research on work–family enrichment. At the same time, the findings also confirm that work–family enrichment mediates the relationship between thriving at work and family role performance. That is, in addition to the direct effect of thriving at work on family role performance, it also indirectly contributes to family role performance by affecting employees’ work–family enrichment. According to the work–home resource model, employees actively use this work resource to spill over the benefits it brings to the family domain, thereby improving their role performance in family activities, i.e., generating work–family enrichment (Ilies et al., 2017; Wood et al., 2020; Wayne et al., 2022). Abid and Contreras (2022) also pointed out that the positive psychological resources of individuals are the main aspect that facilitates the occurrence of work–family enrichment. From the results of the present study, it appears that thriving at work is just such a positive psychological state that promotes the occurrence of work–family enrichment, which in turn has a positive effect on family role performance.

In addition, in today’s world of increasing work–family conflict, a family-supportive supervisor style that focuses on building harmony between the organization and employees is considered a constructive and popular leadership style for employees. Some studies have found that family-supportive supervisor can help employees transition well between work and family by providing them with work resources and modeling work–family balance (Odle-Dusseau et al., 2012; Hammer et al., 2013). This study describes the boundary conditions of the relationship between thriving at work and work–family enrichment in terms of the environment. The results showed that family-supportive supervisor behavior positively moderated between thriving at work and work–family enrichment and also positively moderated the mediating role of work–family enrichment between thriving at work and family role performance, which to some extent validates and extends the above findings that our inclusion of family-supportive supervisor behavior as an environmental resource to help individuals achieve emotional permeability and physical separation between work and family explains the scenarios under which individuals are able to allocate and generate resources more effectively.

### Theoretical significance

First, due to the finiteness of resources, individual work and family roles are sometimes incompatible, resulting in work–family conflict. However, work and family are not always contradictory and conflicting. Although resources are limited, they have enrichment. Thus, resources in different fields can be transferred and utilized in other fields (Booth-LeDoux et al., 2020; Lin et al., 2021). At present, thriving at work is a relatively new construct in organizational behavior, and there is a lack of research on the positive relationship between it and family-level variables from the perspective of employees’ positive psychological resources. Based on the work–home resource model and from the perspective of resource flow, this study reveals how employees accumulate resources in the work environment and apply them in the family environment, discusses the positive impact of thriving at work on family role performance, and points out the potential boundary conditions. This study enriches the theoretical research on the relationship between thriving at work and family role performance.

Second, it makes up for some deficiencies of the current research on work–family enrichment. Currently, although researchers are showing more and more interest in the field of work–family enrichment, most of the existing studies focus on the negative spillover effects (work–family conflict) between work and family (Xin et al., 2018), and research on the antecedent and outcome variables of work–family enrichment is not very mature. Most studies focus on whether the types of resources provided by organizations are conducive to the generation of work–family enrichment. Only a few studies have focused on the important role of personal psychological resources in the workplace (thriving at work) in realizing positive work–family relationships. This study incorporates work–family enrichment into the model framework. It discusses the mechanism of the effect of thriving at work on family role performance. The results also contribute to research in the field of work–family enrichment by further enriching the work–family resources model, namely, to work in the field of psychological resources by work–family enrichment influence in the field of the family. Thus, it serves as a reference for relevant future research.

Third, previous studies on family role performance mostly reveal the influencing factors of family role performance from the perspective of between-individual static, which makes the research results largely affected by common method biases, impacting the persuasiveness of the empirical results (Podsakoff et al., 2003). Due to its unique advantages, the experience sampling method is the most important method for domestic and foreign researchers to reveal the fluctuating nature of the mechanism of psychological phenomena, such as work exuberance, organizational commitment, work performance, and work satisfaction, within a short period from the perspective of within-individual dynamics (Conway et al., 2015; Diestel et al., 2015). However, studies on the short-term fluctuations of family role performance from the perspective of within-individual dynamics are relatively limited (Chen et al., 2014). This study attempts to “capture” the dynamic experience of employees’ family role performance under natural circumstances using the experience sampling method and analyzes the internal dynamic mechanism of the impact of thriving at work on individual employees’ family role performance. It reveals the obvious short-term fluctuation of family role performance and starts from the work–family resource model. It confirms that the daily individual resource changes of employees’ thriving at work affect their daily family role performance through work–family enrichment, thus it improves our understanding of how family role performance is enhanced. In addition, the application of the multi-level experience sampling method helps to systematically and completely explore the co-promotion effect of thriving at work (within-individual variable) and family-supportive supervisor behavior (between-individual variable) on employees’ family role performance.

Fourth, previous studies have suggested that family-supportive supervisor behavior can help improve employees’ work behavior (Bosch et al., 2018). Some studies have found that family-supportive supervisor behavior is beneficial to easing work–family conflict and improving marital satisfaction (Russo et al., 2018). This study examines the moderating effect of family-supportive supervisor behavior on work–family enrichment through a multi-level analysis and further examines the moderated mediating effect of the first stage. This study enriches and deepens previous research that is based on the work–home resource model by revealing that thriving at work is the generator of resources; work–family enrichment is the converter of resources; and family-supportive supervisor behavior is the catalyst of resource generation and transformation, which regulates the generation of thriving at work (individual resources) and the transformation of resources by work–family enrichment. These results indicate that the positive effects of family-supportive supervisor behavior are not only limited to the work domain or the alleviation of work–family conflict but also can promote a positive work state and the fulfillment of employees’ family life roles through work–family enrichment. Moreover, it reveals that the work–home resource model applies to some extent. A supervisor who cares about the needs of employees’ families and lives can make thriving at work play a greater role, but thriving at work would be greatly reduced when the supervisors do not provide family and life support.

### Practical implications

The results of this study also have many implications for managers and employees. First, the research finds that thriving at work can effectively improve employees’ family role performance, which means that enterprises can integrate thriving at work into their human resource management process, pay attention to guiding employees to form the values of active learning and staying active, and publicize the benefits of this sense of vitality to their family life. Employees should also be aware of the positive impact of continuous learning and vitality at work on their families and achieving a win-win situation between family and work.

Second, work–family enrichment links thriving at work and family role performance. Therefore, managers should attach great importance to the mutually beneficial relationship between work and family. On the one hand, they should understand the dual responsibilities of family and work shouldered by employees and advocate a win-win situation between career and family. On the other hand, companies can create opportunities for employees to demonstrate their work roles, allowing them to apply resources from their work field to their family life while remaining active at work.

Third, family-supportive supervisor behavior plays a positive role in thriving at work and family role performance. Therefore, managers need to increase supportive behaviors for employees’ families, such as helping employees solve their family challenges to coordinate the demands of work and family better. Moreover, when selecting and promoting leaders, the organization can focus on leaders who demonstrate more family-supportive behaviors, or the organization can organize various training to cultivate and encourage leaders to form a family-supportive supervisor style, which would not only promote the family role performance of employees but also benefits the organization.

### Limitations and future directions

This study has certain limitations and aspects that need to be further studied. First, this study adopts the experience sampling method. This method captures the dynamic changes between variables to a certain extent and helps deduce causality more effectively (Moskowitz and Young, 2006). However, we can still provide sufficient evidence of the causality between the variables. Moreover, all the data are self-reported, so there may be some common method biases. Therefore, future studies can collect core data at different time points or adopt a more rigorous longitudinal study design supplemented by field experiments to obtain more accurate and effective causal inference.

Second, due to the specific nature of the experience sampling method of data collection, there are greater difficulties in selecting a sample, resulting in a smaller sample size. All the samples of this study are from enterprises and public institutions in a particular area in northwest China, which reduces the influence of regional cultural and economic differences on the research results and restricts the generalizability of the research results. Future research can collect samples from different regions to improve the universality and applicability of the conclusions. In addition, this study covers married employees in various enterprises and institutions in China and does not explore the study population by industry and field. Therefore, it is not clear whether thriving at work has different effects on married employees in different industries and fields. In future research, married employees should be analyzed by industry to provide targeted measures for companies in different industries.

Third, further additions to the moderating variables are needed to build on the present model. This study argues that work–family enrichment is the key to linking thriving at work and family role performance, and thus, it is important to explore how to enhance this relationship. The present model does not consider environmental factors and only incorporates family-supportive supervisor behavior. The impact of factors such as organizational climate and a sense of family support on the relationship between thriving at work and work–family enrichment can be further explored in the future.

Fourth, through argument and hypotheses testing, this study mentions the potential role of some variables but does not measure them. For example, explaining the relationship between thriving at work and family role performance will be conducive to improving employees’ self-efficacy and happiness in life. However, we do not measure self-efficacy and happiness in life. Therefore, researchers can measure these variables in future studies to significantly improve our understanding of the relationship between thriving at work and family role performance.

## Conclusion

Based on the work–home resource model, this study uses an experience sampling method to explore the impact of thriving at work on family role performance and the mechanism of work–family enrichment and family-supportive supervisor behavior. We find that thriving at work positively affects family role performance partly through the mediating effect of work–family enrichment at the individual level. Moreover, family-supportive supervisor behavior moderates the relationship between thriving at work and work–family enrichment. Through work–family enrichment, family-supportive supervisor behavior also moderates the indirect relationship between thriving at work and family role performance. Specifically, the higher the level of family-supportive supervisor behavior, the stronger the indirect effect of thriving at work on family role performance through work–family enrichment.

As demonstrated, work and family are not always in conflict and individuals need to look at things as positively as possible, taking full advantage of their work prosperity and utilizing the positive experiences that work brings at home. Companies should also strengthen their awareness and ability to provide family support behaviors to help employees better differentiate between work and family, isolate themselves from work in time and space, enjoy family life with pleasure and efficiency, and go to work the next day with great anticipation, thus achieving a win–win situation in both the work and family spheres.

## Data availability statement

The original contributions presented in the study are included in the article/Supplementary material, further inquiries can be directed to the corresponding author/s.

## Ethics statement

The studies involving human participants were reviewed and approved by Ethics Committee of the School of Psychology at Northwest Normal University. The patients/participants provided their written informed consent to participate in this study.

## Author contributions

BY was responsible for the research conception, design, and article revision. SS was responsible for article writing, data analysis and translation. ZZ was responsible for researching the feasibility of the article and proposing revisions. QD and JW were responsible for the translation correction and revision of the article. All authors contributed to the article and approved the submitted version.

## Conflict of interest

The authors declare that the research was conducted in the absence of any commercial or financial relationships that could be construed as a potential conflict of interest.

## Publisher’s note

All claims expressed in this article are solely those of the authors and do not necessarily represent those of their affiliated organizations, or those of the publisher, the editors and the reviewers. Any product that may be evaluated in this article, or claim that may be made by its manufacturer, is not guaranteed or endorsed by the publisher.
